# DA^3^4FL: a robust dynamic accumulator-based authentication and key agreement with preserving model training data integrity for federated learning

**DOI:** 10.1038/s41598-025-33685-1

**Published:** 2025-12-28

**Authors:** Songtao Li, Yixuan Zhang, Jie Bai, Kuan Fan

**Affiliations:** 1 Sydney Smart Technology College, Northeastern University, Qinhuangdao, 066006 China; 2 School of Computer and Communication Engineering, Northeastern University, Shenyang, 110819 China

**Keywords:** Federated learning, Authentication and key agreement, eCK adversary, Dynamic accumulator, Blockchain, Engineering, Mathematics and computing

## Abstract

Federated Learning (FL) offers a privacy-preserving distributed learning paradigm by enabling model training without direct access to raw data. However, FL remains vulnerable to unauthorized access during training and client-server exchanges. Authentication and key agreement are essential to restrict access to legitimate participants. Existing FL authentication schemes are prone to impersonation risks, centralized PKI fragility, and insufficient integrity guarantees. To address these challenges, we propose DA$^3$4FL, a robust dynamic accumulator-based authentication and key agreement with preserving data integrity for FL. Specifically, our proposed DA$^3$4FL is an efficient authentication protocol utilizing dynamic accumulators, blockchain technology, and message authentication codes, which ensures robust member management, authorized access, and data integrity. Security analysis against the eCK adversary model confirms the resilience of our protocol. Furthermore, experiments and performance evaluations show the effectiveness of our method, with computational overhead competitive with current state-of-the-art (SOTA) baselines.

## Introduction

Federated Learning (FL) has emerged as a promising machine learning paradigm for distributed model training, preserving the privacy of local raw data^1–3^. Despite its inherent privacy advantages, FL remains susceptible to unauthorized access during transmission phases^4^, posing significant risks of privacy compromise. Essential security measures include mutual authentication to prevent unauthorized access, session key secrecy to safeguard data transmission, and integrity verification to detect model tampering^5^. Among these measures, Authentication and Key Agreement (AKA) plays a crucial role in FL, as it not only verifies the legitimacy of participating entities but also ensures secure and trusted communication. Without effective AKA mechanisms, FL systems are vulnerable to various attacks such as impersonation, man-in-the-middle, and data leakage, which can severely undermine the privacy and integrity of the learning process. Therefore, the design of robust AKA schemes is essential to establish a secure communication environment in FL.

**Fig. 1:**
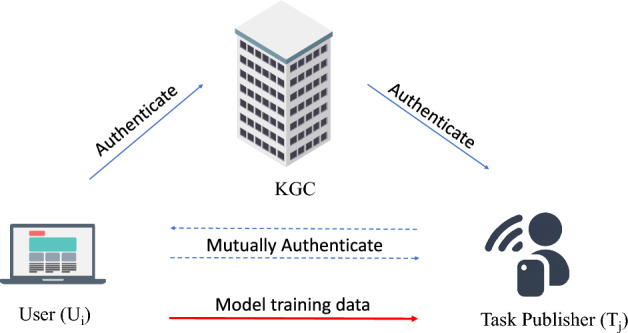
Typical AKA framework in FL.

The standard AKA workflow in FL is depicted in Fig. 1, involving three principal entities: client devices (participants), a central server (coordinator), and a trusted authority (commonly a Key Generation Center, KGC). Specifically, client devices and the server perform mutual authentication through the mediation or assistance of the KGC, followed by the establishment of a session key for secure communication. This session key is subsequently used to encrypt model updates and other sensitive information during the training process, ensuring data confidentiality and integrity against external threats. However, this conventional AKA framework faces two fundamental challenges considering security and performance perspectives.

**Challenge 1: Security weakness:** most existing authentication protocols rely on a centralized Public Key Infrastructure (PKI) framework^6–8^. Should the KGC be compromised or experience downtime, the entire FL system becomes vulnerable. Huang et al.’s protocol for secure data transmission in FL, while innovative, remains susceptible to this centralization issue and impersonation attacks by legitimate users^9^. On the other side, Fan et al.’s DAFL framework attempts to address decentralization in FL but lacks adequate key agreement functionality, compromising data security^10^. Additionally, current AKA solutions do not ensure the integrity of transmitted training data, potentially leading to failures in the FL process^11^.

**Challenge 2: Inefficient dynamic membership management:** existing solutions face performance challenges from inefficient member management mechanisms. The substantial computational overhead required for authentication and key agreement impedes the model training process^12,13^, particularly when frequent member additions or removals occur. Current AKA protocols typically require complete reconstruction of membership credentials during dynamic updates, which is especially problematic in FL where participant turnover is common^14^. While some decentralized approaches have been proposed^15^, they often incur significant computational and storage overhead due to frequent updates, failing to provide an optimal balance between security and efficiency.

To address above-mentioned challenges, we introduce DA$^3$4FL, a robust dynamic accumulator-based authentication and key agreement with preserving data integrity for FL. DA$^3$4FL is an efficient authentication protocol that implements four key security enhancements: (i) robust mutual authentication and decentralized trust, achieved through blockchain-secured pseudonyms, dynamic accumulators to thwart impersonation attacks^16^; (ii) a hybrid KGC architecture, distributing the KGC’s master secret key via Shamir’s (*k*, *n*) threshold scheme and leveraging blockchain for transparent, tamper-proof state management, which mitigates centralized issues. (iii) secure session key agreement, utilizing Elliptic Curve Cryptography (ECC) resistant to key compromise within the eCK model^17^; and (iv) data integrity protection, ensuring the veracity of model updates via MAC-verified methods to protect the integrity of the model training data^18^.

Performance-wise, the dynamic accumulator provides more efficient member management than static schemes^16^, allowing for real-time user additions or removals without needing to reconstruct the entire member list. The use of IPFS further optimizes the system by offloading large model storage from the blockchain, reducing costs and improving scalability. Compared to state-of-the-art methods^9,10,15^, our protocol exhibits superior performance by achieving a robust balance between enhanced security, decentralization, and efficiency.

Beyond existing FL-AKA schemes (e.g.,^9,10^), DA$^3$4FL (i) decentralizes trust via a thresholded KGC with on-chain state for verifiable recovery; (ii) supports efficient dynamic membership through a cryptographic accumulator with constant-time verification; (iii) preserves model-update integrity using lightweight MACs with IPFS off-chain storage; and (iv) enables quick session-key updates without full re-authentication, while remaining provably secure under the eCK model and competitive in cost.

The main contributions of this study are as follows:

- **Robust dynamic accumulator-based and effective AKA protocol**: We introduce DA$^3$4FL, a brand-new AKA protocol tailored for FL. The protocol ensures mutual authentication and secure encryption of model training data. It employs a dynamic accumulator for efficient member management, leverages blockchain technology for decentralization implements a robust recovery mechanism. Specifically, the KGC’s master secret key is distributed via a threshold scheme, and its state is securely backed up on-chain, enabling a new KGC to be selected and the system state to be fully restored. Additionally, the incorporation of Message Authentication Codes (MAC) guarantees the integrity of the model training data.
- **Detailed security analysis**: We provide a provable security proof for the DA$^3$4FL protocol under the eCK model, ensuring the semantic security of the session key. Subsequent heuristic analysis demonstrate the protocol’s robustness against diverse attacks and underscores its critical functionality.
- **Extensive performance analysis**: We conducted extensive experiments and performed a thorough performance evaluation of DA$^3$4FL, establishing that the protocol not only matches but frequently surpasses the performance of existing solutions. Our comparative performance analysis across five most-related authentication schemes demonstrates that DA$^3$4FL maintains competitive computational and communication costs compared to state-of-the-art protocols.

The remainder of this study is organized as follows: section “Related works” reviews the related works. Section “Problem statement” outlines the preliminary work. Section “The proposed protocol” details the proposed authentication protocol. Sections “Security analyses of the proposed protocol” and “Experiments and performance analyses” provides a comprehensive security and performance analysis of DA$^3$4FL. Finally, section “Conclusion and future work” concludes the paper.

## Related works

This section presents a review of the literature on our topic, covering AKA protocols under centralized and decentralized architectures.

### AKA under centralized architecture

The Public Key Infrastructure (PKI) employs centralized Certificate Authorities (CAs) to issue digital certificates, which are crucial for verifying the identities of entities^7^. This mechanism has been extensively adopted across various sectors. For instance, Siddiqui et al.^19^ proposed a dual-CA model to enhance security in cloud IoT systems. Similarly, Azees^20^ and Vijayakumar^21^ have developed PKI-based protocols for anonymous vehicle authentication, and Huang et al.^9^ have incorporated PKI into their FL scheme.

However, a significant drawback of PKI is its vulnerability due to the centralized nature of certificate issuance. A single compromised CA can jeopardize the security of the entire system. Wang et al.^22^ emphasize that centralized PKI systems are particularly susceptible to security breaches if the CA is compromised. Additionally, traditional authentication methods such as Kerberos and OAuth, which heavily rely on central trust servers, may efficiently authenticate users but lack the robustness required to withstand large-scale attacks^23^.

### AKA under decentralized architecture

Some research has explored blockchain-based alternatives to address the vulnerabilities associated with centralized PKI architectures^24,25^. Wang et al.^22^ developed a blockchain-based decentralized authentication method for smart grid edge computing that enhances data security, albeit at the expense of user anonymity. Parameswarath et al.^15^ introduced a user-empowered authentication scheme for electric vehicle charging that leverages decentralized identity and verifiable credentials, though it incurs considerable storage and communication costs. Similarly, Fan^10^ proposed a decentralized scheme for FL that lacks key agreement, compromising the security of private data. In addition, the study by Wang et al.^22^ also addressed the dynamic addition and removal of members, but was found inefficient in managing these changes. Each time a user joins or leaves, substantial overhead is required to update cryptographic keys and verify member credentials, which is particularly costly in FL where member updates are frequent. In response, decentralized solutions have been suggested^10,15^, yet they still incur significant computational and storage overhead due to frequent updates^22,26,27^.

Moreover, in FL, the aforementioned schemes primarily ensure confidentiality but fail to guarantee data integrity. Even though the model training data is encrypted using session keys, it remains vulnerable to interception by attackers who might corrupt the data sent to trainers, potentially disrupting the training and aggregation processes^11^. Earlier methods like checksums, used to verify data integrity, lacked cryptographic robustness as they did not rely on a shared secret key, thus making them prone to forgery and collision attacks^28^. To safeguard message integrity, digital signatures have traditionally been employed to verify the integrity and authenticity of transactions^10^. However, digital signatures entail significant computational and storage demands. Unlike MACs, digital signatures require asymmetric cryptographic operations, which are computationally intensive and less efficient for frequent message authentication^29,30^. Decentralized authentication schemes often rely on digital signatures instead of MACs, adding to the computational load^31^. While this method ensures authenticity, it proves less efficient in resource-constrained environments like FL, where lightweight authentication mechanisms are preferred^32^.

## Preliminaries

This section overviews the core building blocks used in DA$^3$4FL: dynamic accumulators for efficient membership management, the eCK threat model for formal security, message authentication codes (MACs) for lightweight integrity, and Shamir’s (*k*, *n*) threshold secret sharing for KGC resilience. Table 2 summarizes the notation used throughout the paper.

### Dynamic accumulator

This subsection consolidates the dynamic accumulator background used throughout the protocol. The proposed scheme utilizes an accumulator as a repository for registered entities, converting a set containing numerous identities and public key information into an accumulator value while generating a concise witness to verify that a specific element belongs to the set *F*^33,34^. Dynamic accumulators enable the committee to manage member identities without retaining all member information, thus enhancing the efficiency of the authentication scheme. Compared to schemes lacking dynamic accumulators, this scheme provides considerable advantages in computational efficiency and storage costs.

In this scheme, the dynamic accumulator comprises five functions: $Wit(\cdot )$, $Ver(\cdot )$, $Add(\cdot )$, $Del(\cdot )$, and $MemWitUp(\cdot )$. The witness generation function, $Wit(f_i, ACC_F, F)$, produces proof for an element $f_i$ using the accumulator value $ACC_F$ and the set *F*. The verification function, $Ver(f_i, wit_i, ACC_F)$, assesses the validity of the witness. The addition function, $Add(ACC_F, F, f_i)$, and the deletion function, $Del(ACC_F, F, f_i)$, modify the set and accumulator value as elements are added or removed. Additionally, the witness update function, $MemWitUp(ACC_F, f_i, wit_i)$, ensures that the witness for any modified element remains valid, thereby preserving the integrity of the set’s cryptographic proof.

To justify our choice of dynamic accumulators, Table 1 compares DA$^3$4FL’s accumulator-based approach with alternative membership data structures (Merkle trees and Bloom filters) in terms of asymptotic complexity, revocability, false positives, and message rounds required for authentication. As shown, dynamic accumulators offer constant-time operations for add, delete, and verify, support true revocability without false positives, and enable efficient three-round authentication. Merkle trees incur logarithmic overhead for updates and verification, while Bloom filters lack true deletion and introduce probabilistic false positives, making them unsuitable for security-critical AKA protocols.

**Table 1:** Comparison of membership data structures for AKA.

Scheme	Add	Del	Verify	Revocable	False positive	Message rounds
Dynamic Accumulator	$\mathscr {O}(1)$	$\mathscr {O}(1)$	$\mathscr {O}(1)$	Yes	No	3
Merkle Tree	$\mathscr {O}(\log n)$	$\mathscr {O}(\log n)$	$\mathscr {O}(\log n)$	Yes	No	$\ge 2$
Bloom Filter	$\mathscr {O}(1)$	$a \times b$	$\mathscr {O}(1)$	No	Yes	1–2

### Threat model

This subsection centralizes the adversary capabilities assumed in our analysis. To the best of our knowledge, the extended Canetti-Krawczyk model (eCK)^35^ describes an adversary more powerful than those depicted in the Dolev-Yao (DY) model^36^. Such a formidable adversary model presents significant threats to FL. Consequently, it is essential to incorporate the eCK adversary model when assessing the robustness of the proposed protocol. The eCK model specifies eight capacities that the adversary $\mathscr {A}$ may possess.

- $\mathscr {A}$ can intercept, modify, insert, and delete any communication messages in the open channel.
- $\mathscr {A}$ can polynomially offline enumerate all items in the Cartesian product of ID space and password space $S_{ID} \times S_{PW}$.
- To a password-based authentication in the scheme, $\mathscr {A}$ can compromise the following one factor: (a) the user’s password or (b) data stored in the smart card.
- $\mathscr {A}$ can get any previous session key of the user.
- $\mathscr {A}$ can learn the KGC’s secret long-term key when considering the system’s eventual failure.
- $\mathscr {A}$ can obtain ephemeral secrets when testing the security of the session key.
- $\mathscr {A}$ can compromise a user to get his/her sensitive data, and subsequently impersonate the compromised user to participate in the next communication among the KGC, task publisher, and other users.
- $\mathscr {A}$ may be the KGC, only when assessing the security of the user’s password in the registration phase.

### Message Authentication Codes (MACs)

We use MACs for lightweight message integrity and authenticity in FL exchanges. Concretely, an entity computes $MAC = h(TS \parallel SK \parallel M)$ over the timestamp *TS*, the session key *SK*, and the message *M* (e.g., an encrypted model or its hash); the receiver verifies the tag via $Ver(\cdot )$ before acceptance. This aligns with the later “Model upload and Verification” phase and avoids the high cost of digital signatures.

### Shamir’s (*k*, *n*) threshold secret sharing

We employ Shamir’s (*k*, *n*) threshold scheme^37^ to split the KGC master secret among *n* independent holders, ensuring that any *k* shares can reconstruct the secret while fewer than *k* reveal nothing; the detailed process appears later in Algorithm 1 during System Setup.

**Algorithm 1:**
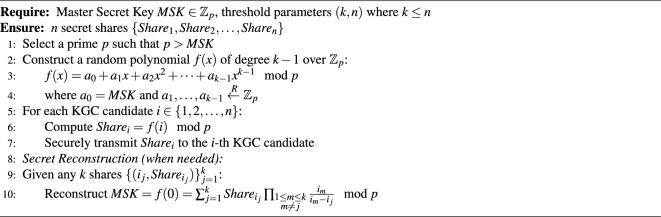
Shamir’s (*k*, *n*) threshold secret sharing.

**Table 2:** Notations of symbols in DA$^3$4FL.

Symbols	Notations	Symbols	Notations
KGC	Key generation center	$h(\cdot )$	Hash function
$U_i$, $T_j$	*i*th user and *j*th task publisher	$PK_i$, $PK_j$	Public key of *i*th user and *j*th task publisher
$x_i$, $x_j$, $x_s$	Private key of *i*th user, *j*th task publisher and KGC	$D_i$, $D_j$	Public dynamic value of *i*th user and *j*th task publisher
$d_i$, $d_j$	Secret dynamic value of *i*th user and *j*th task publisher	$PID_i$, $PID_j$	Pseudo identity of *i*th user and *j*th task publisher
$s_i$, $S_i$	Secret value pair for $U_i$	$s_j$, $S_j$	Secret value pair for $T_j$
*TS*	Time stamp	$\Delta T$	Maximum time to discard a request
*MSK*	Master Secret key of KGC	*SK*	Session key of *i*th user and *j*th task publisher
$ID_i$, $ID_j$	Identity of *i*th user and *j*th task publisher	$PK_s$	Public key of KGC

## Problem statement

### Design goals

The goal of DA$^3$4FL is to design a secure and efficient AKA protocol for FL. Specifically, the objectives are as follows:

1. **Authentication key agreement:** DA$^3$4FL achieves an anonymous AKA process with mitigated centralized issues, ensuring the semantic security defined formally in 6.1.
2. **Protection of local training data integrity:** The protocol ensures the integrity of local training data, preventing any unauthorized modifications or tampering during the FL process. Given *model*, *MAC*, verification is performed using the function:
  $$ V = Ver(model, MAC) $$
3. **Resistance to known attacks:** DA$^3$4FL must comprehensively addresses critical security threats including replay attacks, ephemeral secret leakage, impersonation attacks, and man-in-the-middle attacks and achieves forward secrecy. Our security analysis in section “Heuristic analyses” focuses exclusively on attack vectors relevant to federated learning environments.
4. **Efficient dynamic membership management:** DA$^3$4FL must provide efficient member management than static schemes^16^, allowing for real-time user additions or removals without needing to reconstruct the entire member list.

### System architecture

**Fig. 2:**
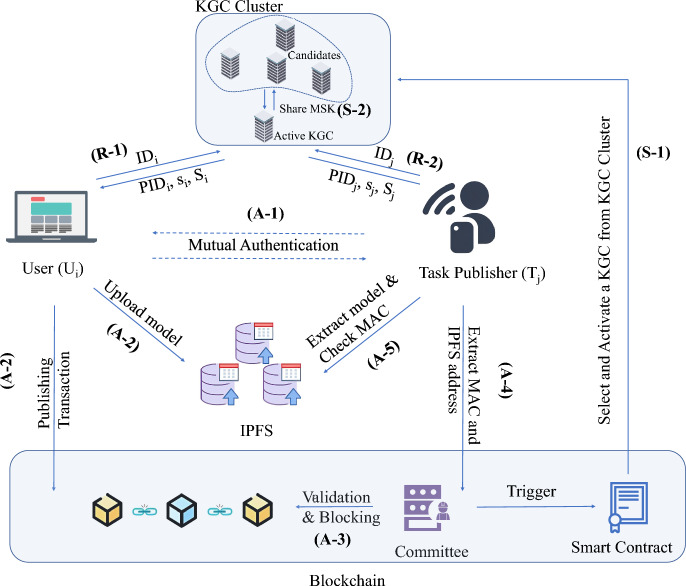
Architecture of DA$^3$4FL.

We introduce the system model of DA$^3$4FL in Fig. 2. It comprises five components: the KGC cluster (with multi-KGC architecture and threshold secret sharing), task publishers (initiating and managing FL training), users (local trainers with data privacy), the blockchain committee (decentralized state management and transaction verification), and IPFS (off-chain storage for large model updates).

**KGC cluster:** The KGC Cluster is structured as a dynamic consortium consisting of one active KGC and $n-1$ candidate KGCs. This cluster is responsible for creating the system’s public parameters and generating pseudonyms and secret values for users and task publishers to facilitate anonymous communication. The active KGC is not a permanent entity. Instead, it is randomly selected from the pool of candidate KGCs by a smart contract on the blockchain at system initialization or during a recovery process triggered by the committee. To ensure the security and resilience of the master secret key, it is securely split into *n* shares using Shamir’s (*k*, *n*) threshold secret sharing scheme, and these *n* shares are distributed among all *n* members of the consortium, meaning no single member possesses the complete master secret key. Furthermore, to facilitate system recovery, critical user state information is encrypted and stored on the blockchain, ensuring it remains accessible even if the active KGC fails.

**Users:** Users act as trainers for local models. They acquire local models through training, perform mutual authentication with task publishers, upload model training data to IPFS, and submit upload requests to the blockchain along with a MAC.

**Task publishers:** Task publishers initiate training requests on the blockchain and negotiate session keys through the authentication process. Once the blockchain validates the reliability of the uploaded models from users, task publishers decrypt the models using the session keys and aggregate them.

**Committee:** The committee operates a consortium blockchain. It handles transaction submission (training requests and model uploads), verifies senders via the accumulator, caches verified transactions, and discards failed ones. Members can be appointed by a trusted organization or elected via a committee algorithm^38^ and reach consensus using an appropriate protocol^39^. If the KGC fails, the committee detects it and triggers recovery through the KGC management smart contract.

**Interplanetary File System (IPFS):** IPFS is utilized to store uploaded models. Due to the inefficiency and high cost of uploading large files directly to the blockchain, this system involves uploading model training data to IPFS and storing the returned addresses on the blockchain. We use IPFS for mature content-addressed storage and broad gateway/tooling support, keeping large payloads off-chain. Swarm and Filecoin are compatible but add tighter Ethereum coupling (Swarm) or storage-market overhead/latency (Filecoin), so we omit them here; backends are swappable without changing DA$^3$4FL’s message flow^40^.

Initially, during the system setup phase, the committee triggers the smart contract to select an active KGC from KGC candidates using a verifiable random function **(S-1, S-2)**. After that, the active KGC generates system parameters and initialize the system. Then, during the registration phase, both users and task publishers register with the active KGC, receiving pseudonyms and secret values while the KGC stores encrypted user states on-chain **(R-1, R-2)**. Subsequently, the user and task publisher authenticate each other through blockchain-verified pseudonyms and negotiate a session key using dynamic accumulators for constant-time membership verification **(A-1)**. Utilizing this session key, the user encrypts model updates and uploads them to IPFS, submitting only the content identifier and MAC to the blockchain **(A-2)**. Following this, the committee verifies the legitimacy of the sender using the accumulator value $ACC_F$ and the task publisher checks MAC integrity **(A-3)**. Finally, the task publisher retrieves encrypted models from IPFS, decrypts them using the corresponding session keys, and aggregates the models **(A-4, A-5)**.

## The proposed protocol

### Main idea

The core of our DA$^3$4FL protocol is built upon four key design ideas. (i) A hybrid architecture integrates a centralized Key Generation Center (KGC) for efficient registration and pseudonym generation with a decentralized blockchain for tamper-proof state backup and transaction verification. (ii) A (*k*, *n*) threshold scheme distributes the KGC’s master secret key to enable verifiable recovery. (iii) A dynamic accumulator enables the blockchain committee to verify membership with constant-time operations, ensuring efficient and scalable management. (iv) Message Authentication Codes (MACs) are used to verify the integrity of encrypted model updates stored off-chain. The protocol encompasses a System Setup Phase, Registration and Authentication Phases for secure participation, a Dynamic Accumulator-based membership management mechanism, a Model Upload and Verification Phase to ensure model integrity, an Update Phase for managing dynamic membership and secret refreshment. This hybrid architecture combines the performance benefits of centralized computation with the fault tolerance and long-term integrity guarantees of decentralization. The meanings of symbols used in the protocol are detailed in Table 2.

### System setup phase

The system setup phase establishes the foundational parameters and initializes the blockchain. This process is orchestrated by a KGC cluster, which consists of *n* candidate KGC nodes, and a blockchain committee. The setup proceeds as follows:

(i) **Blockchain and accumulator initialization**: The blockchain committee collaborates to initialize the consortium blockchain. They first generate the initial set *F*, which includes all committee members and the *n* candidate KGCs. For each entity *k*, an element $f_k = h(PID_k \parallel PK_k)$ is computed. The committee and the candidate KGCs then cooperate to compute the initial accumulator value $Acc_{F} = u^{f_1 f_2 \ldots f_m}$, where $u \in \mathbb {Z}_n^*$ and *m* is the number of initial members. This value $Acc_{F}$, along with the set *F* and a timestamp, forms the genesis block of the blockchain.
(ii) **Active KGC selection**: The dedicated KGC management smart contract is first initialized with the list of n candidate KGCs, according to Algorithm 2. Upon initialization, the contract executes the SelectActiveKGC algorithm using on-chain randomness (e.g., the latest block hash) to verifiably select the first active KGC from the candidate pool. This node assumes the role of the active KGC for the initial phase.
(iii) **Parameter generation by the active KGC**: The newly selected active KGC performs the following steps to generate the system’s core cryptographic parameters: *Key initialization:* The active KGC randomly selects a master private key (*MSK*) and defines the public system parameter (*n*). *Hash function selection:* It chooses a secure one-way hash function $h: \{0,1\}^* \rightarrow \mathbb {Z}^*_n$, sets its identity ($ID_s$), and computes the pseudonymous identifier $PID_s = ID_s \oplus h(PK_s \parallel MSK)$. *Elliptic curve setup:* It selects two large primes (*p* and *q*) that meet the security parameter (*n*), generates an elliptic curve $E(F_p)$ over the prime finite field $F_p$, and chooses a generator *P* of the *q*-order additive subgroup *G* based on $E(F_p)$. Subsequently, it selects its private key ($x_s$) for the blockchain and generates the corresponding public key ($PK_s$). *Parameter publication:* The active KGC securely stores $(MSK, x_s)$ and publishes the system public parameters $\{h, PID_s, q, P, G, PK_s\}$ to all participants.
(iv) **Threshold secret sharing and distribution:** After generating the *MSK*, the active KGC applies Shamir’s (*k*, *n*) threshold secret sharing scheme^41^ to split the master key *MSK* into *n* shares. These shares are then securely distributed to all *n* members of the KGC cluster, ensuring that any subset of *k* members can reconstruct the *MSK*, while any subset of fewer than *k* members gains no information about it. The detailed process is formalized in Algorithm 1.

**Algorithm 2:**
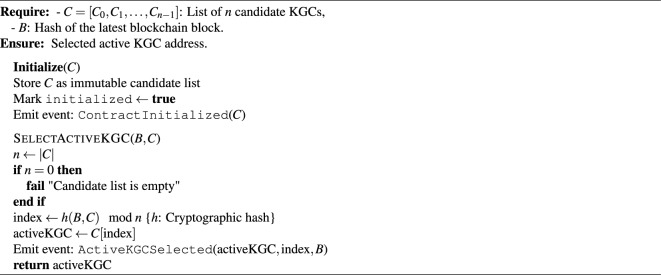
Verifiable KGC selection with initialization.

Upon completion of these steps, the system is fully initialized, and the active KGC is ready to handle subsequent operations such as user registration and authentication. For the remainder of the protocol description, unless otherwise specified, all references to the “KGC” denote the selected active KGC, who is responsible for managing the day-to-day operations of the system. The other members of the KGC cluster are referred to as candidate KGCs.

### Registration phase

First, user $U_i$ locally generates a private-public key pair. $U_i$ randomly selects a 160-bit number $x_i$ as the private key and calculates the corresponding public key $PK_i = x_i P$. $U_i$ then selects a password $PW_i$ and compute $x_i^m = x_i \oplus h(ID_i \parallel PW_i)$. Then, $U_i$ initiates the following registration steps with the KGC.

(i) **Identity selection and initial request:** $U_i$ first randomly chooses an identity $ID_i$ and sends $ID_i, PK_i$ along with the registration request to the KGC.
(ii) **KGC processing and response:** Upon receiving the registration request from $U_i$, $KGC$ selects a secret number $s_i$, then computes $S_i = PK_i + s_i \cdot P$ and computes $PID_i = ID_i \oplus h(PK_i \parallel MSK)$. KGC stores $(S_i, PID_i)$ in list $L_s$ securely. After that, KGC sends $(PID_i, s_i, S_i)$ to $U_i$ via a secure channel. Then, the KGC computes $Ciphertext_i=S_i \oplus h(MSK \parallel PID_i)$ and upload ($PID_i$, $Ciphertext_i$) to the blockchain. Lastly, KGC puts $f_i = h(PID_i \parallel PK_i)$ to the set *F* and updates the accumulator value $ACC_f$ to the blockchain.
(iii) **User response and blockchain update:** $U_i$ receives $(PID_i, s_i, S_i)$. Then $U_i$ randomly selects $d_i \in \mathbb {Z}_q^*$, calculates $D_i = d_i P$ and publishes its $(PK_i, D_i)$ in blockchain. $U_i$ then securely stores $(s_i, d_i, PID_i, x_i^m)$ in a smart card.
(iv) **Witness generation:** $U_i$ generates an existential witness $wit_i = Wit(f_i, ACC_F, F)$ based on the accumulator value $ACC_F$ in the blockchain.
(v) **Committee verification and block addition:** A committee appends $\{PID_i, PK_i, Acc_F\}$ to the most recent block. Other committee members validate $Acc_F$ through $Ver(f_i, wit_i, ACC_F)$; if valid, the block is added, and their witness $MemWitUp(ACC_F, f_i, wit_i)$ is refreshed in the accumulator. Otherwise, the block is discarded.

The registration of task publisher $T_j$ is similar to $U_i$’s. So, it is omitted here.

### Authentication phase

The DA$^3$4FL authentication involves three message rounds: (1) $U_i \rightarrow$ KGC: user initiates authentication with $(M_1, M_2, M_3, M_4, TS)$; (2) KGC $\rightarrow T_j$: KGC forwards verified request with $(M_5, M_6, M_7, M_8, TS)$; and (3) $T_j \rightarrow U_i$: task publisher completes key agreement with $(M_{10}, M_{11})$.

**Fig. 3:**
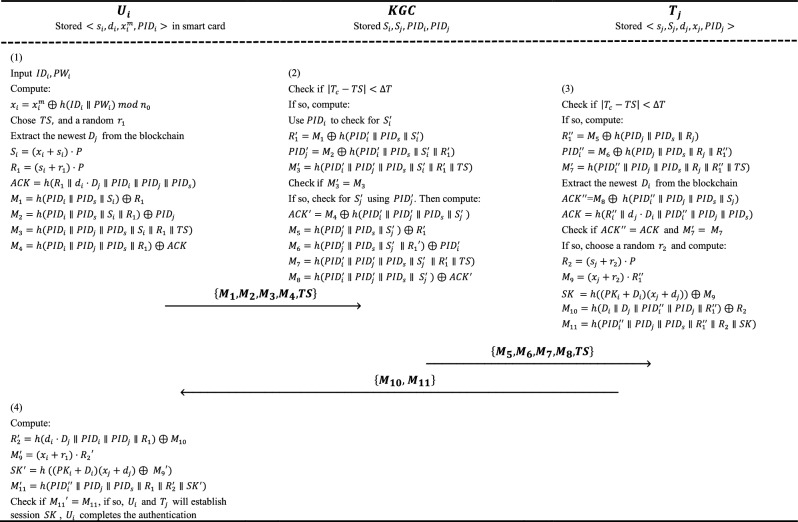
Authentication and session key agreement in DA$^3$4FL.

In this phase, $U_i$ and $T_j$ can share parameters of the model securely after that $U_i$ and $T_j$ authenticate each other. The detailed steps in the authentication phase are shown below. To enhance the understanding of the authentication phase, the scheme’s workflow and detailed cryptographic computation process is illustrated in Fig.  3.

(i) **Identity and password verification:** User $U_i$ enters their identity $ID_i$ and password $PW_i$. The smart card computes $x_i = x_i^m \oplus h(ID_i \parallel PW_i) \mod n_0$, where $n_0$ is the smallest positive integer such that $n_0 G = O$, with *O* denoting the point at infinity on the elliptic curve. It then retrieves $D_j$ from the blockchain using $PK_j$, selects a random nonce $r_1$, records the current timestamp *TS*, and sends the authentication request $(M_1, M_2, M_3, M_4, TS)$ to the KGC over a public channel.
(ii) **KGC time validation and data retrieval:** Upon receiving the authentication request $$(M_1, M_2, M_3, M_4, TS)$$ from $$U_i$$, $$KGC$$ verifies the time validity by checking $$ |T_C - TS| < \Delta T $$, where $$\Delta T$$ is the maximum time threshold for accepting messages, and $$T_C$$ is the current time. If the time is invalid, $$KGC$$ discards the request; otherwise, $$KGC$$ goes to the next step. $$KGC$$ use $$PID_i$$ to check for $$ S_i'$$ and retrieves $$S_i'$$ in the list $$L_s$$. Then $$KGC$$ computes the $$(R_1', PID_j', M_3')$$.
(iii) **Parameter verification and authentication by KGC:** $KGC$ checks the validation of $M_3' = M_3$. If not, $KGC$ discards the authentication request; otherwise, $KGC$ can authenticate $U_i$ and retrieves $S_j'$ in the list $L_s$ through $PID_j'$. Finally, $KGC$ sends message $(M_6, M_7, M_8, TS)$ to $T_j$ by a public channel with $( M_5, M_6, M_7, M_8, TS)$.
(iv) **Time check and data extraction by **$T_j$: Upon receiving the message $(M_5, M_6, M_7, M_8, TS)$ from KGC, $T_j$ initially checks the validation of time by $|T_C - TS| < \Delta T$. If so, $T_j$ extracts $PID_s$ from the public channel and calculates $(R_1'', PID_i'', M_7')$.
(v) **Final validation and message transmission by **$T_j$: $T_j$ validates $M_7' = M_7$. If the validation fails, $T_j$ denies the communication request. Otherwise, $T_j$ can verify KGC and then proceed with the following steps. $T_j$ extracts newest $D_i$ and $D_j$ and then computes $(ACK'', ACK)$. Then $T_j$ validates if $ACK'' = ACK$. If not, $T_j$ discards the communication request. Otherwise, $T_j$ chooses a random number $r_2$ and computes and then transmits message $(M_{10},M_{11})$ to $U_i$.
(vi) **Completion of authentication by **$U_i$: When $U_i$ obtains the message $(M_{10},M_{11})$ from $T_j$, $U_i$ first computes $(R_2',M_{9}'),SK',M_{11}')$ and verifies $M_{11}' = M_{11}$. If so, $U_i$ can authenticate $T_j$ and establish the common session key $SK = SK^{'}$. If not, $U_i$ discards the communication request.

### Model upload and verification phase

After the authentication process, both $U_i$ and $T_j$ share the same session key *SK*. Then $U_i$ can encrypt his/her model training data to *C*, using symmetric encryption algorithms, such as AES. Given that transmitting the local model on the blockchain would significantly increase communication costs, $U_i$ initially stores the encrypted model *C* on IPFS, and the corresponding transaction includes the IPFS address *addr*. Specifically, $U_i$ initiates an upload transaction by calculating $MAC = h(TS \parallel SK \parallel C_{hash})$, where $C_{hash}$ is the hash of the encrypted model *C*. $U_i$ then publishes the transaction $(PID, addr, MAC, TS, wit_i)$ to the blockchain.

To realize a unified workflow that integrates authentication with model transmission, the protocol binds upload immediately to the just-established session. This design mitigates the downsides of sequential decoupling by eliminating a TOCTTOU (Time-of-Check to Time-of-Use) window and preventing re-binding/replay: at upload time the committee verifies membership with $ACC_F$ and $wit_i$, and the task publisher re-checks the MAC using the same *SK*. Freshness via $|T_C-TS|<\Delta T$ and constant-time accumulator verification ensure atomicity with respect to revocation and state changes, while lightweight per-upload session-key updates further reduce exposure without full re-authentication.

Upon receiving transaction $(PID_i, addr, MAC, TS, wit_i)$, the blockchain committee first verifies the timeliness by checking $|T_C - TS| < \Delta T$. The committee then confirms the node’s presence in the Accumulator using $Ver(f_i, wit_i, ACC_F)$. If confirmed, the transaction is added to the block. The task publisher subsequently retrieves the transaction from the blockchain and extracts *C* from IPFS by *addr*. They compute $MAC^{'}=h(TS \parallel SK \parallel C_{hash})$ and checks if $MAC{'}=MAC$. If they match, the model is accepted; otherwise, it is discarded.

### Dynamic update mechanisms

In this update phase, we address the updating of session keys with dynamic update mechanisms, $PID_i$, membership details, and users’ passwords.

#### Update of session keys

In scenarios where each upload of model training data necessitates a distinct session key, traditional approaches typically entail re-authentication and key agreement, thereby imposing substantial communication overhead. Our method introduces an efficient mechanism for updating session keys after initial authentication, significantly reducing associated communication costs.

Upon successful authentication, both the user $U_i$ and the task publisher $T_j$ securely store their respective $M_{9}$ values, timed with $T_m$. During this phase, it is imperative that $T_m$ remains within its effective period to ensure the validity of $M_{9}$. Both parties merely need to generate new random values $d_i^{new}$ and $d_j^{new}$, subsequently publishing $D_i^{new}$ and $D_j^{new}$ on the blockchain. Thereafter, they are able to calculate the new session key $SK = h((x_i + d_i^{new})(PK_j + D_j^{new}) \parallel M_{9})$. For highly sensitive data requiring stringent security, setting $T_m$ to 0 mandates a full re-authentication process instead of utilizing the shortcut for key negotiation and authentication.

#### Update of PID and secret values

To ensure anonymity and untraceability, $U_i$ may request immediate identity updates from the KGC. Specifically, when $U_i$ updates their identity and secret values with the KGC, both entities adopt new identities and secrets as follows: $PID_i^{new} = PID_i \oplus h(S_i \parallel R_1)$. Subsequently, the KGC records the relationship between $PID_i$ and $PID_i^{new}$, and then refreshes the accumulator value using $PID_i^{new}$.

#### Update of membership

Considering the addition of new members, they must adhere to the established registration procedure. For members intending to leave, such as user $U_i$, a resignation transaction is sent to the blockchain. The Committee then executes $Del(U_i)$ to exclude the member from the accumulator set $F$ and updates the accumulator value $ACC_F$ in the subsequent block. Thereafter, the KGC uses the participant’s $PID_i$ to locate $U_i$’s information and deletes it from the list $L_s$.

#### Update of users’ password

$U_i$ can also change his/her password with the following steps:

1. $U_i$ first inputs his/her identity $ID_i$ and password $PW_i$, and the smart card will compute: $x_i = x_i^m \oplus h(ID_i \parallel PW_i)$.
2. $U_i$ selects the new password $PW_i^{new}$ and computes ${x_i^m}^{new} = x_i \oplus h(ID_i \parallel PW_i^{new} \parallel R_1)$.
3. Then he/she stores $({x_i^m}^{new})$ in a smart card securely.

#### Update of active KGC and MSK

The update of MSK begins when a committee member triggers the KGC rotation contract. The contract executes SelectActiveKGC (Algorithm 2) to verifiably elect a new KGC using on-chain randomness.

The newly elected KGC collects the shares from the committee members and reconstructs the previous master secret key *MSK*. Using *MSK*, it recovers the hidden state $S_i$ from the on-chain pseudonym and ciphertext pair via:

$$ S_i = Ciphertext_i \oplus h(MSK \parallel PID_i) $$

After that it processes any backlogged registration requests that are still within their valid time window. Then, the KGC generates a new master secret key $MSK^{\text {new}}$ and re-encrypts the data as:

$$ Ciphertext_i^{\text {new}} = S_i \oplus h(MSK^{\text {new}} \parallel PID_i) $$

and uploads $Ciphertext_i^{\text {new}}$ to the blockchain. Finally, the new KGC generates $MSK^{\text {new}}$ and distributes its (*k*, *n*) Shamir shares securely to the *n* committee members.

Upon completion of these steps, the system resumes normal operation with the new active KGC.

## Security analyses of the proposed protocol

We analyzed the security and performance of the proposed protocol. The security analysis includes: (1) *Provable security*, where we demonstrate the semantic security of the session key in this section; (2) *Heuristic analysis*, which shows that the protocol can resist known attacks. The performance analysis involves the protocol’s functionality, communication and computation, where we compare the proposed DA$^3$4FL with three related schemes.

### Formal security analysis

In this section, based on the eCK adversarial model, we give a security formal proof that the adversary cannot compromise the semantic security of the session key in our proposed protocol.

Note that the security of our scheme fundamentally relies on two well-known hard problems in elliptic curve cryptography. The first is the Elliptic Curve Discrete Logarithm Problem (ECDLP), which posits that it is computationally infeasible for an adversary $\mathscr {A}$ to determine the scalar $a$ given a point $P$ on an elliptic curve and its scalar multiple $aP$, even with knowledge of both $P$ and $aP$^42^. The second is the Elliptic Curve Computational Diffie-Hellman Problem (ECCDHP), which states that, given two points $aP$ and $bP$, where $a$ and $b$ are unknown scalars in $\mathbb {Z}_p^*$, it is computationally infeasible for any polynomial-time adversary to derive the shared key $abP$ with significant probability^9^.

#### Basics for formal proof

In protocol $\mathbb {P}$, there are three participants: the user $U_i$, the task publisher $T_j$, and the KGC. Before initiating the simulation, the simulator begins by selecting an elliptic curve $E(F_p)$ over a prime finite field $F_p$ and chooses a generator *P* from the additive subgroup of order *q*, where *p* and *q* are large prime numbers. The length of *q* meets $|q| = n$. Following this, the user $U_i$ obtains personal information $\{{ID_i, PW_i}\}$, along with a smart card containing {$PID_i, d_i, x_i^m, S_i$}. Meanwhile, the active KGC is selected by the smart contract and it generates the master secret key *MSK*, applies Shamir’s (*k*, *n*) threshold secret sharing scheme to split *MSK* into *n* shares $\{Share_1, Share_2, \ldots , Share_n\}$, and securely distributes each share to *n* independent KGC candidates, and the task publisher $T_j$ stores information $\{{PID_j, d_j, x_j, S_j}\}$ secretly.

In the proof, three entities are instantiated individually as follows: $U_i$ is instantiated as $$\begin{matrix} \prod _{U_i}^u \end{matrix}$$, $T_j$ as $$\begin{matrix} \prod _{T_j}^t \end{matrix}$$, and KGC as $$\begin{matrix} \prod _{KGC}^k \end{matrix}$$. When we want to represent any of them, we can simplify their notation to $\prod _{}^t$. Each instance is treated as an oracle, meaning that its state will change from accept, reject, or return “$\bot$” (indicating no response) depending on whether the input message is valid, invalid, or null.

Following, we provide some terms used in this proof.

${Acceptance\, State }$. When instance $$\begin{matrix} \prod _{}^t \end{matrix}$$ successfully receives the final expected communication in the protocol, it enters an acceptance state. During this session, all transmitted and received messages are sequentially concatenated to generate the session identifier for instance $$\begin{matrix} \prod _{}^{t} \end{matrix}$$.

${Partnering }$. Instances $$\begin{matrix} \prod _{}^{t_1} \end{matrix}$$ and $$\begin{matrix} \prod _{}^{t_2} \end{matrix}$$ are regarded as matched when the following conditions achieve that: (a) both are in an acceptance state, (b) they authenticate each other using the identical session identifier.

${Adversary }$. The eCK adversary $\mathscr {A}$ is presumed to interact with the simulator and honest parties via oracle queries. $\mathscr {A}$ tries to compromise the integrity of authentication messages or the confidentiality of the session key by leveraging the oracle responses. The types of queries that $\mathscr {A}$ is allowed to perform are outlined below.

- $\textit{Execute}$($$\begin{matrix} \prod _{U_i}^u, \prod _{KGC}^k, \prod _{T_j}^t \end{matrix}$$). In this query, $\mathscr {A}$ emulates the full authentication procedure and captures the communications of $U_i$, KGC, and $T_j$.
- $$Send $$($$\begin{matrix} \prod _{}^{t}, m \end{matrix}$$). Using this query, $$\mathscr {A}$$ can transmit message *m* to instance $$\begin{matrix} \prod _{}^{t} \end{matrix}$$ to initiate an attack, with the simulator replying by protocol $$\mathbb {P}$$.
- $$SessionKeyReveal $$ ($$\begin{matrix} \prod _{}^{t} \end{matrix}$$). Through this query, $$\mathscr {A}$$ obtains previous session keys from its corresponding partner instance.
- $$EphemeralKeyReveal $$ ($$\begin{matrix} \prod _{}^t \end{matrix}$$). This query grants the adversary $$\mathscr {A}$$ access to temporary keys, such as nonce values or short-term secret data.
- $$Corrupt $$($$\begin{matrix} \prod _{U_i}^u \end{matrix}, \delta $$). Through this query, $$\mathscr {A}$$ can retrieve critical data stored on the user’s smart card.
- $$Corrupt $$($$\begin{matrix} \prod _{KGC}^k \end{matrix}$$). This query allows $$\mathscr {A}$$ to obtain the master secret key *MSK* along with the private key $$x_s$$.
- $$Corrupt $$($$\begin{matrix} \prod _{T_j}^t \end{matrix}$$). Using this query, $$\mathscr {A}$$ can access confidential information belonging to $$T_j$$.

${Freshness }$. An instance, whether $$\begin{matrix} \prod _{U_i}^u \end{matrix}$$, $$\begin{matrix} \prod _{KGC}^k \end{matrix}$$, or $$\begin{matrix} \prod _{T_j}^t \end{matrix}$$, is considered to be fresh if $\mathscr {A}$ has not gained access to the session key between $U_i$ and $T_j$.

${Test }$($$\begin{matrix} \prod _{}^{t} \end{matrix}$$). This query is designed to assess the semantic security of the session key *SK*, and $\mathscr {A}$ is allowed to execute this query only once. In the context of protocol $\mathbb {P}$, the instance $$\begin{matrix} \prod _{}^{t} \end{matrix}$$ can either represent $$\begin{matrix} \prod _{U_i}^u \end{matrix}$$ or $$\begin{matrix} \prod _{T_j}^t \end{matrix}$$. If the instance $$\begin{matrix} \prod _{}^{t} \end{matrix}$$ has already been queried by ${Test }$($$\begin{matrix} \prod _{}^{t} \end{matrix}$$), then the query will return “$\perp$” (null). Otherwise, the oracle will flip a fair coin *b*. If $b=1$, ${Test }$($$\begin{matrix} \prod _{}^{t} \end{matrix}$$) provides the actual session key *SK* to $\mathscr {A}$; otherwise, it returns a random string with the same length as *SK*.

${Semantic \, Security }$. In protocol $\mathbb {P}$, a PPT adversary $\mathscr {A}$ can perform several queries including *Execute*, *Send*, *Compromise*, and *Test*. $\mathscr {A}$ aims to guess the outcome of the coin flip *b* in the *Test* query and submits its guess $b^*$. Let $Succ(\mathscr {A})$ represent the probability that $\mathscr {A}$ correctly guesses $b^*$. The adversary’s advantage in breaking the semantic security of the session key in protocol $\mathbb {P}$ is defined as:

$$\begin{aligned} Adv_{\mathbb {P}}^{\mathscr {A}}=2{\textrm{Pr}}[\textit{Succ}(\mathscr {A})] -1 \end{aligned}$$

#### Semantic security proof

Following, we give a detailed security proof to indicate the advantage of adversary breaking the session key’s semantic security can be negligible.

##### **Theorem 1**

*Let* $\mathbb {P}$ *represent the proposed protocol, and* $Adv_p^{ECDLP}(n)$ *and* $Adv_p^{ECCDHP}(n)$ *represent the probability advantage of a PPT adversary* $\mathscr {A}$ *in solving the ECDLP and ECCDHP, respectively. If a PPT adversary* $\mathscr {A}$ *carries out* $q_e$ **Execute**, $q_s$ **Send**, $q_h$ **Hash**,* then the advantage that* $\mathscr {A}$ *has in compromising* $\mathbb {P}$ *is negligibly:*

$$Adv_{\mathbb {P}}^{\mathscr {A}} \le \frac{2q_h^2+6q_s}{2^{l_1}}+\frac{(q_s+q_e)^2}{p}+2(C'q_{send}^{s'} +Adv_p^{ECDLP}(n)+Adv_p^{ECCDHP}(n)) $$

##### *Proof*

In the step-by-step proof, a sequence of games is defined from **Game**$_{\textbf {1}}$ to **Game**$_{\textbf {9}}$. Let $Succ_i$ denote the likelihood that $\mathscr {A}$ successfully guesses the *b* in *Test* query in **Game**$_{{\boldsymbol{i}}}$, for $\{i=1,2,\dots ,9\}$.

**Game**$_{\textbf {1}}$: This game models a real-world attack scenario under the random oracle model. A bit *b* is chosen at the beginning. Therefore:

1 $$\begin{aligned} Adv_{\mathbb {P}}^{\mathscr {A}} = 2\textrm{Pr}[Succ _1] - 1 \end{aligned}$$

**Game**$_{\textbf {2}}$: This game introduces a hash table $\Omega _h$. Whenever $\mathscr {A}$ makes a hash query $h(\gamma )$, the hash oracle $\Theta _h$ looks for $\gamma$ in the table $\Omega _h$. If the value $h(\gamma )$ is already stored, $\Theta _h$ returns it. If not, it creates a random value $\psi$, and sends it to $\mathscr {A}$, and records the pair $(\gamma , \psi )$ in $\Omega _h$.

In this scenario, $\mathscr {A}$ tries to use the available hash list to issue the *Test* query, aiming to figure out whether the session key is real or randomly generated. Factually, the session key $SK = h((x_i + d_i)(PK_j + D_j) \parallel M_{10})$ is derived using secret elements like $x_i$, $r_1$, $x_j$ of $T_j$, $r_2$, and random numbers $d_i$ and $d_j$. Without knowledge of these secret components, $\mathscr {A}$ cannot calculate *SK* and is left to guess, making it impossible to determine if $b=0$ or $b=1$ with certainty.

As a result, the probability that $\mathscr {A}$ can succeed in **Game**$_{\textbf {2}}$ by eavesdropping is no greater than its advantage in **Game**$_{\textbf {1}}$, meaning:

2 $$\begin{aligned} \textrm{Pr}[Succ _1]=\textrm{Pr}[Succ _2] \end{aligned}$$

**Game**$_{\textbf {3}}$: In this game, adversary $\mathscr {A}$ attempts to deceive one of the participants into accepting a fabricated message by issuing multiple *Send* and *Hash* queries. Unlike **Game**$_{\textbf {1}}$ and **Game**$_{\textbf {2}}$, $\mathscr {A}$ might gain an increased advantage by finding collisions. If the following collision events occur, the game is terminated:

(i) A collision in the hash outputs, occurring with a probability of $\frac{q_{h}^2}{2^{l+1}}$, where *l* represents the bit length of the hash output.
(ii) Collisions between the random values ($d_i$, $d_j$, $r_1$, $r_2$) may occur with a probability of $\frac{(q_{d_i}+q_{d_j})^2}{2p}$.

Thus, we have:

3 $$\begin{aligned} |{\textrm{Pr}}[{Succ}_3]-{\textrm{Pr}}[{Succ}_2]|\le \frac{{q}_{h}^2}{2^{l+1}}+\frac{({q}_{d_{i}}+{q}_{d_{j}})^2}{2{p}} \end{aligned}$$

**Game**$_{\textbf {4}}$: In this game, adversary $\mathscr {A}$ attempts to guess the values of $M_3$, $M_7$, *ACK*, and $M_{11}$ without making any hash queries.

It follows that:

4 $$\begin{aligned} |\textrm{Pr}[Succ _4]-\textrm{Pr}[Succ _3]|\le \frac{q _s }{2^{l_1 }} \end{aligned}$$

**Game**$_{\textbf {5}}$: Here, adversary $\mathscr {A}$ tries to deduce the value of $M_{10}$ without issuing any hash queries.

Similarly, we obtain:

5 $$\begin{aligned} |\textrm{Pr}[Succ _5]-\textrm{Pr}[Succ _4]|\le \frac{q _s }{2^{l_1 }} \end{aligned}$$

**Game**$$_{\textbf {6}}$$: In this game, adversary $$\mathscr {A}$$ executes a lost smart card attack by issuing a $$Corrupt $$($$\begin{matrix} \prod _{U_i}^u \end{matrix}$$) query. With the help of the “fuzzy keyword and honeyword” approach, the probability of $$\mathscr {A}$$ guessing the correct user password is no more than $$C'q_{send}^{s'}$$ , where constants $$C'$$ and $$s'$$ relate to the $$\mathscr {D}$$^43^. In this case, $$\mathscr {A}$$ makes up to $$q_{send}$$ attempts within the password space $$\mathscr {D}$$. Therefore, we derive:

6 $$\begin{aligned} |\textrm{Pr}[Succ _6]-\textrm{Pr}[Succ _5]|\le C '  q _{send }^{s '} \end{aligned}$$

**Game**$$_{\textbf {7}}$$: Here, adversary $$\mathscr {A}$$ interacts with the oracles $$EphemeralKeyReveal $$($$\begin{matrix} \prod {}^t \end{matrix}$$) and $$SessionKeyReveal $$($$\begin{matrix} \prod _{}^t \end{matrix}$$) to obtain outdated session keys $$SK_{outdated}$$ and random values $$d_i$$, $$d_j$$. Nonetheless, without knowledge of the secrets $$x_i$$ and $$s_i$$, $$\mathscr {A}$$ is unable to compute $$M_{10}$$. Alternatively, $$\mathscr {A}$$ might attempt to find a hash collision.

Hence, we have:

7 $$\begin{aligned} |\textrm{Pr}[Succ _7]-\textrm{Pr}[Succ _6]|\le \frac{q _h ^2}{2^{l_1 +1}} \end{aligned}$$

**Game**$$_{\textbf {8}}$$: In this scenario, $$\mathscr {A}$$ issues a $$Corrupt $$($$\begin{matrix} \prod {T_j}^t \end{matrix}$$) query to access the task publisher $$T_j$$’s secret values, such as $$x_j$$ and $$s_j$$. Despite successfully compromising $$T_j$$, adversary $$\mathscr {A}$$ is unable to derive $$d_j$$ from $$D_j$$ or $$r_2$$ from $$R_2$$, as solving the ECDLP in polynomial time remains infeasible for $$\mathscr {A}$$.

That is, we can have:

8 $$\begin{aligned} |\textrm{Pr}[Succ _8]-\textrm{Pr}[Succ _7]|\le Adv _p ^{ECDLP }(n ) \end{aligned}$$

**Game**$_{\textbf {9}}$: In this game, adversary $\mathscr {A}$ is no longer allowed to use the *Execute*, *Send*, or *Corrupt* queries and instead tries to calculate the session key directly. Since there is no efficient algorithm to solve the ECCDHP, $\mathscr {A}$’s advantage in recomputing $r_1 \cdot r_2$ to retrieve $M_{10}$, denoted as $Adv_{p}^{ECCDHP}(n)$, is negligible.

Therefore, we conclude:

9 $$\begin{aligned} |\textrm{Pr}[Succ _9]-\textrm{Pr}[Succ _8]|\le Adv _p ^{ECCDHP }(n ) \end{aligned}$$

At this stage, the probability of $\mathscr {A}$ successfully guessing the correct session key does not significantly exceed $\frac{1}{2}$, meaning that $\textrm{Pr}[Succ _9]=\frac{1}{2}$.

By equations (1) through (9) and leveraging the triangle inequality, we get

$$\begin{aligned} \begin{aligned} Adv_{\mathbb {P}}^{\mathscr {A}}&= 2 \Pr [Succ_1] - 1 \\&= 2 \Pr [Succ_9] - 1 + 2 (\Pr [Succ_1] - \Pr [Succ_9]) \\&\le \frac{2 q_h^2 + 6 q_s}{2^{l_1}} + \frac{(q_{d_i} + q_{d_j})^2}{p} + \Delta , \end{aligned} \end{aligned}$$

where

$$\begin{aligned} \Delta = 2 (C' q_{send}^{s'} + Adv_p^{ECDLP}(n) + Adv_p^{ECCDHP}(n)). \end{aligned}$$

In conclusion, adversary $\mathscr {A}$ does not hold a significant advantage $Adv_\mathbb {P}^{\mathscr {A}}$ in breaking the semantic security of session key *SK*. The advantage is bounded by the following expression: $Adv_\mathbb {P}^{\mathscr {A}}$ to break the semantic security of *SK*, where $Adv_{\mathbb {P}}^{\mathscr {A}}\le \frac{2q_h^2+6q_s}{2^{l_1}}+\frac{(q_{d_i}+q_{d_j})^2}{p}+2(C'q_{send}^{s'} +Adv_p^{ECDLP}(n)+Adv_p^{ECCDHP}(n))$. $\square$

### Heuristic analyses

This section offers a comprehensive analysis of the proposed scheme’s security features, which include mutual authentication, session key establishment, and forward secrecy. Additionally, it illustrates the scheme’s efficacy in mitigating various attacks, such as replay attacks, password guessing, and man-in-the-middle attacks. We also executed simulations for four representative attacks–replay, impersonation, man-in-the-middle (MITM), and ephemeral secret leakage (ESL)–and observed fast rejection in all cases: replay 0.068 ms (± 0.017), impersonation 0.249 ms (± 0.039), MITM 0.495 ms (± 0.010), and ESL 1.797 ms (± 0.037). Note that the experiments were conducted on a virtualized computing environment based on an AMD EPYC 9754 128-Core Processor. The virtual machine allocated for the experiments provided 8 CPU cores and 16 GB of RAM, running on Ubuntu 24.04.2 LTS.

#### Mutual authentication

Mutual authentication is the process where two parties verify each other’s identities. In the proposed DA$^3$4FL, the KGC authenticates $U_i$ by verifying $M_3$. Meanwhile, $T_j$ authenticates the KGC by verifying $M_7$ and authenticates $U_i$ by verifying $ACK$. Additionally, $U_i$ authenticates $T_j$ by verifying $M_{12}$, thereby achieving mutual authentication.

#### Session key establishment

The session key agreement ensures that no party can compute the session key on its own or predict it in advance without cooperation from others. Factually, the session key $SK = h((x_i + d_i)(PK_j + D_j) \parallel M_{9})$ relies on contributions from $U_i$’s ($d_i$, $x_i$, $r_1$) and $T_j$’s ($PK_j$, $D_j$, $R_1$), ensuring that neither $U_i$, KGC, nor $T_j$ can independently derive the session key.

#### Conditional privacy protection

Conditional privacy protection ensures that under normal circumstances, user privacy is maintained through anonymity and identity protection. However, in the event of malicious activity, the KGC can trace and reveal the true identity of the user. The KGC assigns pseudonyms $PID_i = ID_i \oplus h(PK_i \parallel MSK)$ to each node for transactions, where *MSK* is divided into *n* shares and distributed among multiple independent committee nodes using Shamir’s (*k*, *n*) threshold secret sharing scheme. To uncover true identities, an adversary would need to compromise at least *k* out of *n* committee nodes to reconstruct *MSK*, which is considered practically infeasible. This ensures effective privacy protection. Each pseudonym $PID_i$ is valid for a specific authentication, and after completing an upload operation, the pseudonym will be a new pseudonym $PID_i^{\text {new}} = h(PID_i \parallel S_i \parallel R_1)$. Linking $PID_i$ and $PID_i^{\text {new}}$ requires simultaneous access to $MSK$, $S_i$, and $R_1$, ensuring that pseudonyms from the same source remain unlinked.

#### Forward secrecy

Forward secrecy ensures that, even if long-term secrets such as the KGC *x* and *MSK* are compromised, previous session keys remain secure. Consider a scenario where an adversary knows *x*, *MSK*, $x_i$, $x_j$, $R_1$ and $R_2$. Despite this knowledge, given the complexity of the ECCDHP, the adversary cannot compute $r_1 \cdot r_2$ to derive the session key *SK*, thereby preserving the protocol’s forward secrecy.

#### Data integrity of model updates

Data integrity means that the task publisher accepts only the exact ciphertext bytes produced by the authenticated client within the current freshness window. To forge a model update, an adversary must produce a valid on-chain message $(PID_i, addr, MAC, TS, wit_i)$. However, the adversary cannot forge the MAC, because the message authentication code is computed as $\text {MAC} = h(TS \parallel SK \parallel C_{\text {hash}})$, which requires the session key *SK*. Therefore, data integrity is preserved.

#### Password guessing attacks

As defined in^44^, password-guessing attacks can be classified into two categories: those targeting validation data stored in the smart card (Attack I) and those exploiting verification data transmitted over a public channel (Attack II). In Attack I, the smart card stores only password-related values such as $x^m$, which leaves no accessible verification data for attackers to exploit. In Attack II, the password verification process relies on *ACK*. Even if attackers acquire $S_i$ and retrieve *ACK* by accessing the smart card, they cannot verify guessed passwords or identities ($PW_i$ or $ID_i$) because the modular operations in $x_i$ ensure security.

#### Replay attacks

Replay attacks involve an attacker attempting to resend old messages to pass verification. However, in each session, $U_i$ and $T_j$ generate new random values, $d_i$ or $d_j$, ensuring the uniqueness and freshness of the messages. Consequently, the attacker cannot reuse previous messages or compute the session key to bypass $U_i$’s verification.

#### Man-in-the-Middle (MITM) attack

In a Man-in-the-Middle (MITM) attack, an adversary intercepts the communications between the user and $T_j$, capturing the messages $(M_1, M_2, M_3, M_4, TS)$ sent by the user and the responses $(M_{10}, M_{11})$ from $T_j$. The adversary may attempt to forge a new set of messages $(M_1^*, M_2^*, M_3^*, M_4^*, TS^*, M_{10}^*, M_{11}^*)$ or replay old ones. However, they are unable to convince $U_i$ or $T_j$ that they hold the secret values $S_i$, $S_j$, or the private keys $x_i$ and $x_j$. Consequently, the attacker fails to pass the authentication process, effectively preventing the MITM attack.

#### Ephemeral secret leakage (ESL) attack

This type of attack, also known as a transient key leakage attack^35,45^, occurs when an attacker attempts to derive the session key by intercepting temporary session data, such as the random values $d_i$ and $r_1$. In our proposed DA$^3$4FL, the session key *SK* is produced by combining both short-term random values and long-term secret data, including $x_i$ and $s_i$. Consequently, even if an attacker obtains $d_i$ and $r_1$, they cannot compute the session key *SK*, rendering the attack ineffective (Table 4).

**Table 3:** 10 Criteria for evaluating authentication schemes.

Category	ID	Criteria	Definition
Ideal attributes	I$_1$	Password friendly	Users can freely select and locally modify their passwords
	I$_2$	Sound repairability	Users can join dynamically, and smart card can be revoked
	I$_3$	Key agreement	Users and task publishers must establish a session key after authentication
	I$_4$	Mutual authentication	All parties should authenticate each other’s identities
	I$_5$	No password verifier table	Only users store their password-related data
Security attributes	S$_1$	User anonymity	Adversaries cannot deduce or track users’ identities
	S$_2$	No password exposure	Privileged participants (e.g., KGC administrators) cannot access user passwords during registration
	S$_3$	Forward secrecy	Even if KGC’s long-term key is compromised, the session key remains secure
	S$_4$	Resistance to known attacks	The protocol withstands impersonation, MITM, replay, stolen verifier, and DoS attacks
	S$_5$	Resistance to smart card loss attack	The protocol remains secure even if a smart card is lost

**Table 4:** Security functionalities comparisons of all related authentication schemes.

Schemes	Year	Criteria									
		I$_1$	I$_2$	I$_3$	I$_4$	I$_5$	S$_1$	S$_2$	S$_3$	S$_4$	S$_5$
Huang et al.^9^	2024	$\surd$	$\surd$	$\surd$	$\surd$	$\surd$	$\surd$	$\surd$	$\surd$	$\times$	$\surd$
He et al.^46^	2023	$\surd$	$\surd$	$\surd$	$\surd$	$\times$	$\times$	$\times$	$\surd$	$\times$	$\times$
Fan et al.^10^	2023	$\times$	$\surd$	$\times$	$\surd$	$\surd$	$\surd$	$\surd$	$\surd$	$\times$	—
Parameswarath et al.^15^	2022	—	$\surd$	$\times$	$\surd$	—	$\surd$	—	$\times$	$\times$	—
Srinivas et al.^47^	2020	$\surd$	$\surd$	$\surd$	$\surd$	$\surd$	$\surd$	$\surd$	$\surd$	$\times$	$\surd$
DA^3^4FL	**—**	$\surd$	$\surd$	$\surd$	$\surd$	$\surd$	$\surd$	$\surd$	$\surd$	$\surd$	$\surd$

#### Impersonation attack

For an external malicious node, lacking the private key and long-term secret values of a legitimate node, it cannot impersonate a legitimate entity to communicate with the KGC. For a legitimate user, computing the private key and long-term secret values of the KGC during the session key negotiation process is infeasible due to the ECDLP, thereby preventing impersonation of the KGC.

## Experiments and performance analyses

In this section, we present a comprehensive performance analysis, including functionality comparisons and cost estimation comparisons among five state-of-the-art (SOTA) user authentication protocols. Note that the experiments were conducted on a virtualized computing environment based on an AMD EPYC 9754 128-Core Processor. The virtual machine allocated for the experiments provided 8 CPU cores and 16 GB of RAM, running on Ubuntu 24.04.2 LTS.

**Table 5:** Comparisons of computation and communication costs among authentication protocols.

Schemes	Year	Computational cost			Total communication cost : bits
		User	Task publisher	KGC	
$\mathrm{Huang\ et\ al.}$ ^9^	2024	$2T_{sp} + 7T_h$	$2T_{sp} + 7T_h$	—	800
$\mathrm{He\ et\ al.}$ ^46^	2023	$5T_{sp} + 6T_h$	$5T_{sp} + 7T_h$	—	1782
$\mathrm{Fan\ et\ al.}$ ^10^	2023	$T_{sp} + 2T_h$	$3T_{sp} + 2T_h$	—	1472
$\mathrm{Parameswarath\ et\ al.}$ ^15^	2022	$T_{vsig} + 7T_h$	$3T_h + T_{vsig}$	$6T_h + T_{gsig}$	7872
$$\mathrm{Jangirala\ et\ al.}$$ ^47^	2020	$$10T_h + 4T_{sp} $$	$$16T_h + 5T_{sp}+ T_{fe}$$	$$9T_h + 2T_{sp}$$	2656
DA$^{ {3}}$4FL	—	$9T_h + 5T_{sp}$	$8T_h + 4T_{sp}$	$8T_h$	2164

### Functionality analysis

To evaluate the strengths and weaknesses in terms of functionality, the widely recognized 10 criteria^48^ were employed. These criteria comprise five ideal attributes (*I*) and five security attributes (*S*), as detailed in Table 3. In terms of ideal attributes, the DA$^3$4FL outperforms existing schemes in several key areas. For password friendliness ($I_1$), unlike Fan et al.’s scheme which does not permit users to modify their passwords locally^10^, both Huang et al. and the proposed protocol support this feature^9^. Additionally, He et al.’s scheme^46^ also supports local password modification, but Srinivas et al.’s scheme^47^ enhances this by integrating robust password management mechanisms without compromising usability. Regarding key agreement ($I_3$), Parameswarath et al.’s scheme^15^ lacks the capability to establish a session key, and He et al.’s scheme^46^ does not provide mutual authentication ($I_5$), potentially limiting its practicality in certain contexts. All schemes except Parameswarath et al.’s^15^ offer mutual authentication ($I_4$). Furthermore, Parameswarath et al.’s use of a password verifier table ($I_5$) poses a risk of password leakage^15^, while He et al.’s scheme^46^ does not fully safeguard mutual authentication, leaving it susceptible to specific attacks.

**Table 6:** The length of all terms.

Symbols	Bits	Symbols	Bits
module ($n_0$)	32	time stamp (*t*)	32
ECC point (*p*)	256	random/nonce (*r*, *d*)	160
hash value (*h*)	160	user’s ID (*ID*)	128
public key (*PK*)	512	private key (*x*)	256
password (*PW*)	160	secret value (*s*, *S*)	160

In terms of security attributes, the proposed protocol, along with other schemes, ensures user anonymity ($S_1$), preventing adversaries from identifying or tracking users. Regarding password exposure ($S_2$), schemes by Huang et al.^9^, Fan et al.^10^, and the proposed protocol prevent administrators from accessing users’ passwords during registration, a feature missing in Parameswarath et al.’s scheme^15^. However, He et al.’s scheme^46^ fails to adequately protect against password exposure during registration. The proposed protocol also supports forward secrecy ($S_3$), safeguarding past session keys even if the long-term key is compromised, a feature absent in the schemes of Parameswarath et al. and Srinivas et al.^15,47^. Regarding resilience to known attacks ($S_4$), Huang et al.’s scheme^9^ cannot defend against impersonation attacks, nor can Parameswarath et al.’s and Srinivas et al.’s schemes^15,47^ defend against ESL attacks. For $S_5$, He et al.’s scheme^46^ cannot effectively resist the smart card loss attacks. In contrast, the proposed DA$^3$4FL protocol effectively addresses these vulnerabilities, providing robust protection against known attacks.

**Table 7:** Running time of different cryptographic operations.

Symbol	Description	Time (ms)
$T_{\text {sp}}$	256-bit ECC scalar point multiplication	0.065
$T_{\text {fe}} \approx T_{\text {sp}}$	Biometric-fuzzy extractor	0.065
$T_h$	Hash Operation Time	0.004
$T_{\text {gsig}}$	Group Signature Generation Time	0.445
$T_{\text {vsig}}$	Group Signature Verification Time	0.042

**Table 8:** Comparison of total computational costs per authentication for different schemes.

Authentication schemes	Total computational cost (ms)
Huang et al.^9^	0.316
He et al.^46^	0.702
Fan et al.^10^	0.276
Parameswarath et al.^15^	0.593
Srinivas et al.^47^	0.920
DA$^3$4FL	0.653

### Cost analysis

In this subsection, we conduct a performance analysis comparing communication and computation costs to demonstrate the efficiency of the proposed DA$^3$4FL. To assess communication costs, the lengths of each parameter are detailed in Table 6. Table 5 presents a comparison of communication costs across all evaluated schemes. The proposed DA$^3$4FL achieves a total communication cost of 2164 bits, approximately 72.5% lower than that of Parameswarath et al.^15^, indicating a significant enhancement in communication efficiency. Although our communication cost is higher than that of Fan et al.’s^10^ and Huang et al.’s^9^ schemes, the DA$^3$4FL offers improved security features. Notably, our protocol introduces an efficient method to update session keys without completing the entire authentication process, reducing the average cost to levels comparable to the aforementioned schemes.

Compared to He et al.’s scheme^46^, the DA$^3$4FL incurs a slightly higher communication cost (2164 bits versus 1782 bits). However, He et al.’s scheme lacks essential security features such as user anonymity (S$_1$) and resistance to known attacks (S$_4$), making it less suitable for secure FL environments. Srinivas et al.’s scheme^47^, with a communication cost of 2656 bits–approximately 22.8% higher than our protocol–also falls short in computational efficiency and security robustness, lacking features like forward secrecy (S$_3$) and resistance to smart card loss attacks (S$_5$). Our protocol effectively balances the increased communication cost with stronger security, ensuring robust protection against potential attacks in FL settings.

As shown in Tables 7 and 8, DA$^3$4FL incurs a total computational cost of 0.653 ms. This represents a 7.0% reduction compared to He et al.’s scheme (0.702 ms)^46^ and is approximately 29.0% faster than the protocol of Srinivas et al. (0.920 ms)^47^, thereby demonstrating superior efficiency in latency-sensitive federated learning scenarios. Although our cost exceeds those reported by Parameswarath et al. (0.593 ms)^15^, Huang et al. (0.316 ms)^9^, and Fan et al. (0.276 ms)^10^, respectively–DA$^3$4FL compensates for this modest overhead by integrating additional lightweight cryptographic operations and enabling session-key updates without full re-authentication, thereby enhancing overall security and robustness.

In summary, DA$^3$4FL achieves a favorable trade-off between performance and security, rendering it suitable for resource-constrained federated learning environments. By delivering lower latency than the schemes of He et al. and Srinivas et al. ^47^, while providing more comprehensive security features than the faster yet less feature-rich protocols of Huang et al. ^9^, Fan et al. ^10^, and Parameswarath et al. ^15^, DA$^3$4FL offers a practical and secure authentication solution for federated learning.

### Dynamic accumulator performance evaluation

We conducted a comprehensive performance evaluation of our dynamic accumulator under varying system scales. Specifically, we measured both the latency of three fundamental operations (member addition, witness creation, and membership verification) and the peak memory consumption during accumulator operations, while scaling the number of users from 100 to 10,000. Each measurement was repeated 50 times to ensure statistical significance, and we report both the average and standard deviation in Table 9.

**Table 9:** Dynamic accumulator performance metrics with different user counts.

Users	Add (ms/u)	Witness creation (ms)	Membership verification (ms)	Peak memory (KB)
100	12.29 (± 6.75)	33.09 (± 6.34)	11.49 (± 6.34)	27.11 (± 10.43)
500	12.18 (± 7.36)	33.74 (± 9.72)	11.97 (± 9.75)	64.22 (± 0.02)
1,000	12.30 (± 7.61)	33.05 (± 6.81)	11.34 (± 6.83)	88.08 (± 0.02)
2,000	12.23 (± 7.63)	32.82 (± 5.47)	11.68 (± 5.46)	231.88 (± 0.02)
5,000	12.11 (± 7.39)	34.28 (± 8.51)	12.85 (± 8.50)	877.22 (± 0.02)
10,000	12.16 (± 7.35)	33.27 (± 5.96)	11.62 (± 5.94)	998.59 (± 0.02)

As shown in Table 9, the dynamic accumulator demonstrates excellent scalability and efficiency across both computational and memory dimensions. From a latency perspective, the average time for adding a new member remains consistently stable in the range of 12.1–12.3 ms per user, with minimal fluctuations. This demonstrates nearly linear performance even as the number of users increases from 100 to 10,000. The witness creation latency remains stable around 32–35 ms, with only minor variations, and does not show any significant increase as the number of nodes grows. Similarly, the membership verification latency consistently stays within the range of 11–13 ms, with standard deviations generally within acceptable bounds, indicating consistently efficient verification operations.

From a memory perspective, the memory utilization grows sub-linearly with respect to the number of members, remaining under 1,000 KB even at 10,000 users. This demonstrates exceptional memory efficiency, as the accumulator maintains a compact cryptographic representation of the entire membership set without storing individual member records. The extremely low variance across iterations (on the order of 0.02 KB for most scales) indicates highly stable and predictable memory behavior.

Overall, these performance metrics validate the $\mathscr {O}(1)$ time complexity of the system’s core cryptographic operations and demonstrate minimal storage overhead. Even under dynamic stress tests with user counts scaling up to 10,000, the system maintains low and stable latency with sub-linear memory growth. This confirms that DA$^3$4FL is well-suited for resource-constrained environments and large-scale federated learning deployments, where frequent participant changes and efficient membership management are critical requirements.

## Implementation

For practical deployment, use widely supported 128-bit security defaults (ECC P-256, SHA-256 as the hash *h*, HMAC-SHA-256 for MACs, and AES-CTR for encrypting the model ciphertext *C* with random IVs using an encrypt-then-MAC construction), keep a tight but realistic timestamp window ($\Delta T\!\approx$ 1–5 minutes with clock sync), pin IPFS content across multiple nodes, and choose Shamir’s threshold (*k*, *n*) by balancing collusion resistance and availability: pick *k* so that $f < k \le n - u$, where *f* is the maximum tolerated number of compromised custodians and *u* the maximum expected offline custodians; in practice, $k{=}3/5$ for $n{=}5$, $k{=}4/7$ for $n{=}7$, and $k{=}7/13$ for $n{=}13$ offer balanced trade-offs, while larger *k* (e.g., $\approx 2n/3$) hardens security at the cost of a higher recovery quorum.

Integration with mainstream FL frameworks (e.g., TensorFlow Federated and PySyft) is straightforward because DA$^3$4FL sits beneath the learning logic as a thin authentication layer. Clients run the AKA handshake once per training session to derive *SK*, then attach to each update a message containing $(C, TS, PID_i, MAC)$–where *C* is the ciphertext of the serialized model delta, *TS* enforces bounded freshness, $PID_i$ provides conditional anonymity, and $MAC{=}h(TS\parallel SK\parallel C_{hash})$ binds integrity to the ciphertext. The client-side hook executes before serialization/transmission (hashing, encryption, and MAC computation); the aggregator-side hook executes on receipt (timestamp check, MAC verification, accumulator membership verification when enabled), and only then decrypts before handing the plaintext update to the framework’s standard aggregator. In practice this can be implemented as gRPC/HTTP middleware or message pre/post-processors, wrapping client-update RPCs in TensorFlow Federated and tensor/state send/receive paths in PySyft, without changing model formats, optimizers, or aggregation code; DA$^3$4FL treats updates as opaque bytes.

## Conclusion and future work

AKA is crucial for FL, as it prevents unauthorized data leakage and enhances data security. To overcome the limitations of existing AKA technologies, we introduces a robust DA$^3$4FL protocol utilizing blockchain, Message Authentication Code (MAC), and dynamic accumulator technologies. The DA$^3$4FL design ensures identity verification within FL, as well as the security and integrity of messages, and facilitates dynamic and efficient membership management. Security analysis confirms that DA$^3$4FL is robust against the attack vectors analyzed in this work and secures session keys. Performance analysis demonstrates that DA$^3$4FL strikes an optimal balance between security and performance, making it well-suited for FL environments. Following, future work will focus on exploring the application of DA$^3$4FL in more complex FL scenarios, such as mobile FL environments. Additionally, we plan to investigate the integration of emerging technologies, such as homomorphic encryption and differential privacy, to further enhance the security and privacy of DA$^3$4FL in FL applications. While the present ECC and symmetric-based instantiation is not quantum-resistant, future work will explore quantum-resilient techniques such as quantum-resilient accumulators to provide end-to-end PQ security.

## Data Availability

No datasets were generated or analysed during the current study.
